# Greatly Enhanced Photovoltaic Performance of Crystalline Silicon Solar Cells via Metal Oxide

**DOI:** 10.3390/nano8070505

**Published:** 2018-07-07

**Authors:** Lingling Zhou, Lufei Xiao, Hai Yang, Jie Liu, Xibin Yu

**Affiliations:** 1 Department of Food and Environmental Engineering, Chuzhou Vocational and Technical College, Chuzhou 239000, China; zhoulingling2018@gmail.com (L.Z.); tangshiyong001@gmail.com (L.X.); 2 The Education Ministry Key Laboratory of Resource Chemistry and Shanghai Key Laboratory of Rare Earth Functional Materials, Department of Chemistry, Shanghai Normal University, Shanghai 200234, China; benjaejong@gmail.com

**Keywords:** silicon solar cells, semiconductors, electron–hole pairs

## Abstract

Band-gap alignment engineering has now been extensively studied due to its high potential for application. Here we demonstrate a simple route to synthesize two metal oxide layers and align them together according to their bandgaps on the surface of crystalline silicon (c-Si) solar cells. The metal oxide layers not only extend absorption spectrum to generate extra carriers but also more efficiently separate electron–hole pairs. As a consequence, the photovoltaic performance of SnO_2_/CdO/Si double-layer solar cell (DLSC) is highly improved compared to the controlled Si solar cell, CdO/Si and SnO_2_/Si single-layer solar cells (SLSCs). Via alignment engineering, the SnO_2_/CdO/Si DLSC produces a short circuit photocurrent (*J_sc_*) of 38.20 mA/cm^2^, an open circuit photovoltage (*V_oc_*) of 0.575 V and a fill factor (FF) of 68.7%, a conversion efficiency (*η*) of 15.09% under AM1.5 illumination.

## 1. Introduction

Solar cells have been developing for more than five decades. From the very first generation solar cell to the latest one, the conversion efficiency of the solar cell itself has been largely improved. The update conversion efficiency of GaInP/GaAs solar cells have broken 40% [1,2,3,4]. However, these high efficiency solar cells still have many disadvantages, such as stability issues and high cost, so that they can hardly be put into large-scale production. At this point, with the combination of high purity, natural abundance, a matching insulator, and maturity of production [5,6], crystalline silicon (c-Si) solar cells show their unique advantageous properties. However, there still are several defects of c-Si solar cells, such as optical loss, recombination, and thermal or quantum losses. Among them, optical loss and recombination are deemed to be the two most vital factors. Many efforts have been made by researchers to solve these problems. Silicon nanowires [7,8,9], ZnO nanowires [10], and CuO nanoleaves [11] are some of those extraordinary attempts. The power conversion efficiency has been improved through light trapping enhancement and photocarrier collection facilitation [12,13]. The TiO_2_ half-prolates light trapping schemes were designed to optimize photonic elements on top of unstructured planar absorber layers in the Si cells’ front [14]. An Al_2_O_3_ nanopatterned point structure was used to achieve the rear passivation [15]. It has opened up new opportunities to achieve higher energy conversion efficiency at lower fabrication costs.

Although many textured structures have been made to enhance absorptance of c-Si solar cells, c-Si is still an indirect band-gap semiconductor with a bandgap of 1.12 eV, which is able to utilize only a small fraction of the solar spectrum. Currently, the tandem solar cell is considered to be one of the most promising approaches of the third generation solar cells to solve this problem. It is fabricated with two or more sub-cells which are tandemed according to their bandgaps [16]. The overall open circuit voltage (*V_oc_*) is the sum of those from all individual sub-cells, while the current is the same as that in a single sub-cell once their currents are matched [17,18,19]. As a result, tandem solar cell have broadened absorption spectrum effectively, so that they can tackle absorption and thermalization losses simultaneously by absorbing the higher energy photons, improving the conversion performance.

By using the concept of tandem solar cells for reference, band-gap alignment engineering has been developed for various applications, such as quantum dot solar cells through band alignment engineering [20]. Here we applied CdO and SnO_2_ semiconductors to c-Si solar cells. CdO is one of the transparent conducting oxides (TCOs) that has moderate band-gap and high electrical conductivity. Because of high mobility, CdO is also believed to have large potential for the use in active electronic devices [21,22]. SnO_2_ has wide bandgap of 3.5–4.0 eV and terrific electronic conductivity. It has a great prospect in solar cells. SnO_2_ nanostructures were used as the buffer layer of Sb_2_Se_3_ solar cell [23]. In our research, we have tried to synthesize different nano-structured films of CdO and SnO_2_ semiconductors on the surface of c-Si substrate, respectively. To comparatively investigate the electrical properties and the photovoltaic performance, CdO/Si single-layer solar cell (SLSC), SnO_2_/Si SLSC, and SnO_2_/CdO/Si double-layer solar cell (DLSC) were prepared. The controlled Si solar cell without any deposition was also prepared as a reference. The comparison of the controlled cell, two SLSCs, and SnO_2_/CdO/Si DLSC demonstrates the impact of different layers with different bandgaps on the absorption region and the charge-transfer efficiency. As a result, the short-circuit current (*J_sc_*) and the open circuit voltage were improved, leading to the enhancement of power conversion efficiency (PCE). SnO_2_/CdO/Si DLSC offers broad-band light harvesting and super high minority carrier lifetime. We believe that this cost effective technology can be easily applied in the industrial scale production of Si solar cells.

## 2. Materials and Methods

### 2.1. Materials and Methods

Slices (3.0 × 3.0 cm^2^) of c-Si wafers without Si_3_N_4_ antireflection layer were applied in this work. The thickness of wafers is ~200 μm with a bulk *p-n* junction. Before growing any film on the surface of Si wafer, it was cleaned by rinsing with double distilled water and ethanol to eliminate any impurities. Firstly, two depositing solutions were prepared as solution A and B. Solution A is Cd(CH_3_COO)_2_ solution with the concentration of 0.05 M. In solution B, the concentration of SnCl_4_ is 0.01 M, and the 1:1 ethanol:water mixture is as solvent to prohibit hydrolysis. In order to grow CdO layer on the surface of Si wafers, the dried Si wafer was immersed in solution A for 2 min and was dried again in a drying oven at 80 °C. This procedure was repeated for over four times to ensure the CdO seeds were fully distributed on the silicon’s surface. The deposited Si wafer was thermal treated at 500 °C for 3 min, then the CdO/Si was completed. SnO_2_/Si was synthesized through a spin-coating method. In this step, 0.05 mL of 0.01 M Stannic chloride aqueous ethanol solution were dropped onto Si surface under the speed of 1500 rpm for 40 s, and this operation was conducted five times to ensure the Sn-precursor fully covered on the substrate. The deposited Si wafer was treated under 900 °C in muffle furnace for 5 min to form SnO_2_ film. Combining the above two procedures, SnO_2_/CdO/Si was prepared. SnO_2_ was deposited on the surface of as-synthesized CdO/Si composited cell and anneal at 900 °C for 5 min. For electrochemical analysis, two electrodes were made on the above three deposited Si wafers. Ag grids were then screen-printed on front side, followed by rapid thermal annealing. Chemical vapor deposition (CVD) was employed to deposit Al on the reverse side of the c-Si substrates.

### 2.2. Characterization

Field emission scanning electron microscopy (FESEM HitachiS-4800, Hitachi High-Technologies GLOBAL, Tokyo, Japan) was used to evaluate the morphology and contents of the layers. The absorption spectra were measured on a Cary500 UV–vis–NIR spectrophotometer (Hitachi High-Technologies GLOBAL, Tokyo, Japan) using BaSO_4_ as a reference. The minority carrier lifetime of the samples was measured using the Si wafer life-time SEMILAB WT-2000 PVN (SEMILAB, Budapest, Hungary). The current density–voltage (*J-V*) characteristics of the solar cells were measured using a Zennium electrochemical workstation (Zahner elektrik GmbH & Co.KG, Thüringer Str. 12 96317 Kronach, Germany) under 100 mW cm^−2^ calibration which is performed using a Class A AM 1.5 G spectral distributed Sun 2000 Solar Simulator (Abet Technologies, Inc., Milford, CT, USA). The external quantum efficiency were conducted by using a QTest Station (CROWNTECH, Inc., Indianapolis, IN, USA). The impedance measurements were performed on an external potentiostat (XPOT) of an electrochemical Zennium workstation (Zahner elektrik GmbH & Co.KG, Kronach, Germany) with a frequency ranging from 100 KHz down to 0.1 Hz at forward bias potentials between 0 and 0.5 V (with a 5 mV sinusoidal AC perturbation). The resulting impedance spectra were analyzed with ZSipWin software (v3.10, EChem Software, eDAQ Inc., Colorado Springs, CO, USA).

## 3. Results and Discussion

**Figure 1:**
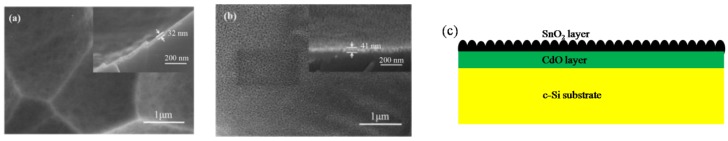
Cross-section and top views of SEM morphologies of (**a**) CdO; and (**b**) SnO_2_ films growing on polycrystalline silicon substrates, respectively, and the cross-sectional views are the inset; (**c**) schematic of SnO_2_/CdO/Si DLSC.

Figure 1 shows the surface morphologies of both CdO and SnO_2_ films deposited on the surface of polycrystalline silicon with *p-n* junction, respectively. As shown in Figure 1a, CdO film was characterized by scanning electron microscope (SEM). The cross-sectional view indicates that the thickness of the film is about 32 nm, and its surface is smooth with comparing top view pictures. While in Figure 1b, SnO_2_ film shows a different surface morphology with many hollows, and it is approximately 41 nm in thickness, slightly thicker than CdO film. With those pictures we can find that both two films have a firm contact with Si substrates, which is beneficial in reducing contact resistance and electron transfer. Figure 1c demonstrates the schematic of SnO_2_/CdO/Si DLSC. With spin-coating method and annealing treatments, CdO layer and SnO_2_ layer were grown on Si substrate in sequence. On one hand, the top layer with a rough surface of SnO_2_ can efficiently increase light trapping. On the other hand, two layers are capable of utilizing utilize different regions of incident light.

**Figure 2:**
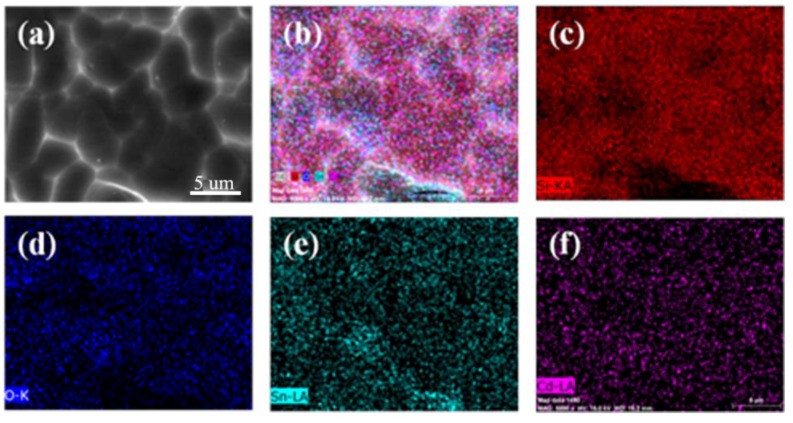
(**a**) FESEM of the composited SnO_2_/CdO/Si DLSC , (**b**) the element mapping of all detected elements, (**c**) Si, (**d**) O, (**e**) Sn, and (**f**) Cd elements.

FESEM energy dispersive spectrometry (EDS) element mapping of composited SnO_2_/CdO/Si DLSC was conducted to take deep research on the structure of layer-by-layer. Figure 2a is the scanning region of SnO_2_/CdO/Si composited wafer, while Figure 2b is the element mapping of all detected elements. Figure 2c–f refer to element Si, O, Sn, and Cd, respectively. From Figure 2c–f, we can find that the elements are well-distributed on the surface of c-Si substrate which indicates that the layer-by-layer structure was greatly formed by spin-coating and annealing procedures.

**Figure 3:**
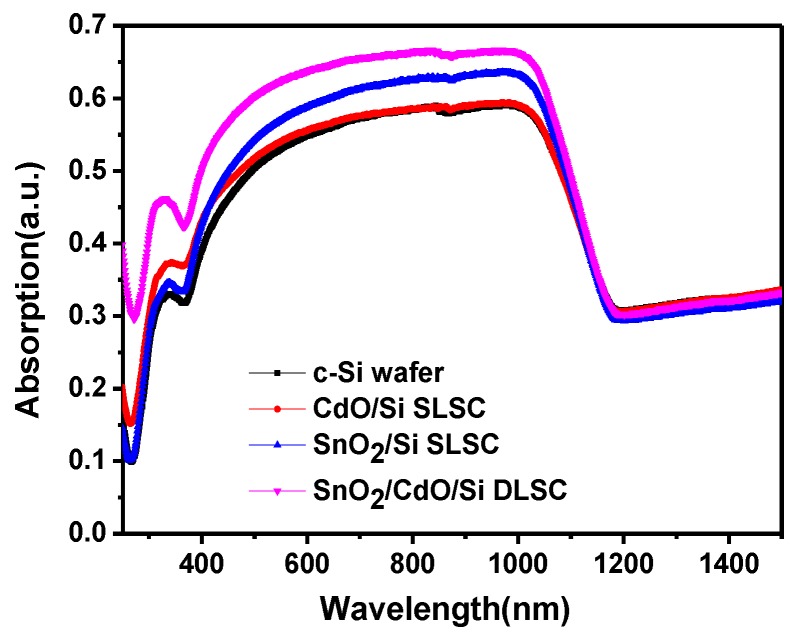
UV–vis–IR absorption spectra for Si wafer, CdO/Si, SnO_2_/Si SLSCs, and SnO_2_/CdO/Si DLSC.

According to different morphologies above, different absorption spectra were measured by UV–vis–IR. In Figure 3, Si substrate with CdO layer shows almost the same absorption property as Si wafer except the slight improvement between 300 and 450 nm. As to SnO_2_ layer, the absorption value from 300 to 1100 nm is improved by about 16%. This phenomenon results from their different surface structures. Because of the hollows on the surface, the film can cause optical resonance and multiple scattering of the incident light, which can trap incident light and enhance absorptance effectively [24,25,26]. In addition, due to its wide bandgap, SnO_2_ film is capable to utilize much more light compared to c-Si wafer. In addition, the SnO_2_/CdO film coatings have an antireflection effect on the solar irradiation. The SnO_2_/CdO coatings can reduce the reflections from the Si wafer surfaces and then increase the absorption. Therefore, the SnO_2_/CdO DLSC has the best absorption in the range of 300–1000 nm.

**Figure 4:**
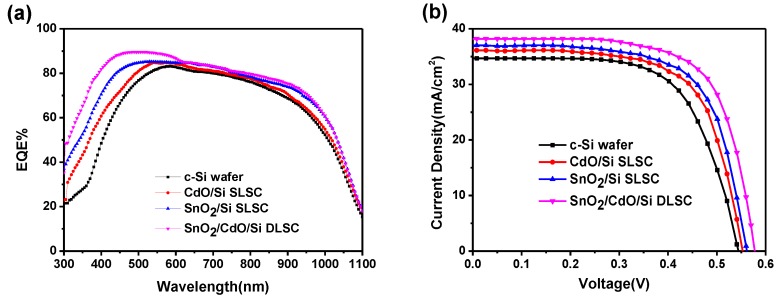
(**a**) External quantum efficiency (EQE) spectra and (**b**) *J-V* characteristics for blank c-Si solar cell, CdO/Si, SnO_2_/Si SLSCs, and SnO_2_/CdO/Si DLSC.

To further investigate photovoltaic properties of composited solar cells, external quantum efficiency (EQE) spectra and *J-V* curves were conducted under the standard AM 1.5 G conditions. Figure 4a shows that a broad value of 70–80% in the spectrum of pristine Si solar cell is observed from 500–900 nm which corresponds to the wavelength range of Si light absorption. In contrast, the spectrum of SnO_2_/Si SLSC exhibits a large increase in EQE spectrum for the wavelength between 300–550 nm, while CdO/Si SLSC is a little lower than that of SnO_2_/Si SLSC. On one hand, as two semiconductors with wide bandgaps (Eg_CdO_ = 2.4 eV, Eg_SnO2_ = 3.5 eV), CdO and SnO_2_ can effectively absorb spectrum wavelengths between 300 and 600 nm. That means the CdO layer and SnO_2_ layer are able to use incident light from 300 to 600 nm and generate more high energy carriers. On the other hand, according to UV–vis–IR absorption spectra, both CdO/Si SLSC and SnO_2_/Si SLSC have been enhanced between 300 and 600 nm. As we know, if solar cells absorb more incident light, more photons can be utilized to generate carriers, so the improvement of EQE can be also attributed to the enhancement of absorption performance. Since the absorption in the range of 800–1000 nm is increased due to the antireflection effect and the hollow structure of SnO_2_ layer, the EQE in the range of 800–1000 nm is increased. Though the absorption in the range of 600–800 nm is also increased, the sensitivity of Si cell to the 600–800 nm irradiation is not as good as that to the 800–1000 nm irradiation. The EQE increase is not obvious in in the range of 600–800 nm. Finally, the EQE performance of SnO_2_/CdO/Si DLSC matches the theory we discussed above as well.

**Figure 5:**
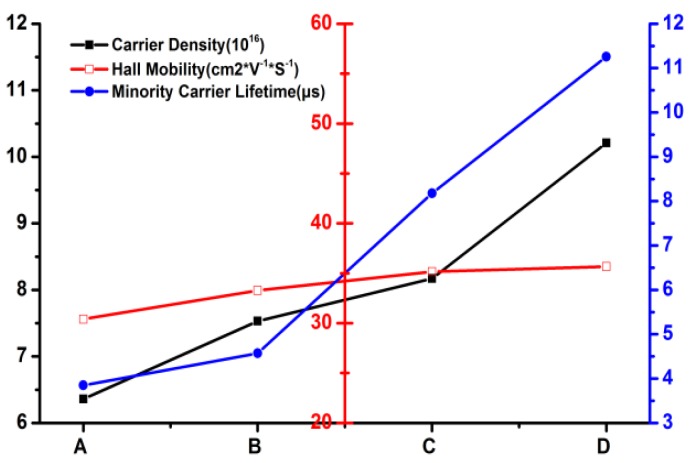
Carrier density, Hall mobility, and minority carrier lifetime properties of (**A**) c-Si solar cell, (**B**) CdO/Si SLSC, (**C**) SnO_2_/Si SLSC, and (**D**) SnO_2_/CdO/Si DLSC.

The detailed device performance is summarized in Table 1 and the *J-V* characteristic curves are shown in Figure 4b. As a result, the PCE of CdO/Si SLSC reaches a value as high as 13.34%, with *J_sc_* of 36.12 mA cm^−2^, *V_oc_* of 0.556 V and FF of 66.42%, while values of SnO_2_/Si SLSC are 13.96%, 37.01 mA cm^−2^, 66.76%. Compared to pristine Si solar cell without any metal oxide film growing on the top, the *V_oc_* performance are improved by 2.6% and 4.2%. Whereas as for current density, three as-synthesized solar cells all have various increases compared to the controlled Si solar cell. The short-circuit currents are mainly related with three factors as we concluded, and the first one is light absorption. The photoactive layer in the solar device absorbs sunlight, raising an electron from the ground state to a higher energy state and then generates an energy bearing electron–hole pair, called an excition [27]. As we know, c-Si is an indirect band-gap semi-conductor with a band-gap of 1.12 eV and the perfect light spectra region for absorption of c-Si is approximately 700 ~ 1100 nm which is only a small part of the whole solar spectrum. In order to maximize *J_sc_*, it is necessary to make full use of the solar spectrum. The more solar spectrum is utilized, the more photo-generated carriers are produced. However, a large part of these photo-generated carriers are wasted directly through recombination. It indicates that if electron–hole pairs can be efficiently separated before recombination, *J_sc_* value is supposed to be enhanced to a large extend. In addition, to ensure an efficient collection of charge carriers, carrier transporting layers are required to have high mobility as well as long diffusion lengths for electrons and holes [28,29,30], and the resistance is also a factor affecting current flowing. In our research, *J_sc_* of CdO/Si and SnO_2_/Si solar cells are increased to 36.12 mA cm^−2^ and 37.01 mA cm^−2^. The increase can be ascribed to absorption enhancement as shown in Figure 3a and charge carrier lifetime increase as shown in Figure 5.

In Figure 5, we can also see that carrier density values of three kinds of as-synthesized solar cells are all enhanced. As we discussed above, because of good solar spectrum absorption property and band-gap alignment engineering, the composited solar cells are able to make better use of solar spectra and thus produce more photo-generated carriers. Carrier density is an important parameter to measure the utilized efficiency of absorbed photons. CdO owns a wider bandgap than c-Si, so short wavelength irradiation is fully used according to the results of UV–vis–IR absorption and EQE property shown in Figure 3. For the same reason, as bandgap of SnO_2_ is even wider, the EQE and UV–vis–IR results indicate the carrier density enhancement which is correspondent with that measured in Figure 5. Moreover, Hall mobility is highly related to electrical conductivity. The result in Figure 5 (red line) shows that the speed of carrier transport is not decayed but improved instead, due to the CdO layer and SnO_2_ layer deposited on top of c-Si substrates. This phenomenon demonstrates that CdO layer and SnO_2_ layer we have synthesized play a role as good electron transport layers. When CdO and SnO_2_ were made to grow on surface of c-Si substrates by a spin coating method, homogeneous films were produced through tuning spinning speed and coating time. After annealing at 500 °C and 900 °C respectively, firm Ohmic contact thin oxide films and c-Si substrates were formed, which is supposed to decrease the series resistance. A blue line is put to demonstrate the change of minority carrier lifetime. The result shows that, after being covered by different oxide layers, the lifetime of the minority carrier is highly increased. The effective carrier lifetime (τ_eff_) is directly related to the bulk lifetime (τ_bulk_) and the surface life time (τ_surf_) and the τ_bulk_ dominates the τ_eff_ [31]. The increase in *J_sc_* and *V_oc_* partly resulted from improved minority carrier lifetime can be understood by the relations below [32]

(1) $$V_{OC}=\frac{kt}{q}\ln(\frac{\Delta n(N_{D,A}+\Delta n)}{n_{i}{}^{2}})$$

(2) $$J_{sc}=qG(L_{n}+L_{p})$$

where kt/q is the thermal voltage, $N_{D,A}$ is the donor or acceptor concentration of the wafer, Δn is the excess carrier concentration, $n_{i}$ is the intrinsic carrier concentration, q is the magnitude of the electrical charge on the electron, G is the generation rate, and $L_{n}$ and $L_{p}$ are electron and hole diffusion lengths, respectively. We can find that *V_oc_* and *J_sc_* strongly depend on excess carrier concentration and diffusion lengths which are directly proportional to the τ_eff_. That is to say, the increase of minority carrier lifetime gives rise to the enhancement of *J_sc_* and *V_oc_*, which in the end contribute to power conversion efficiency.

**Scheme 1:**
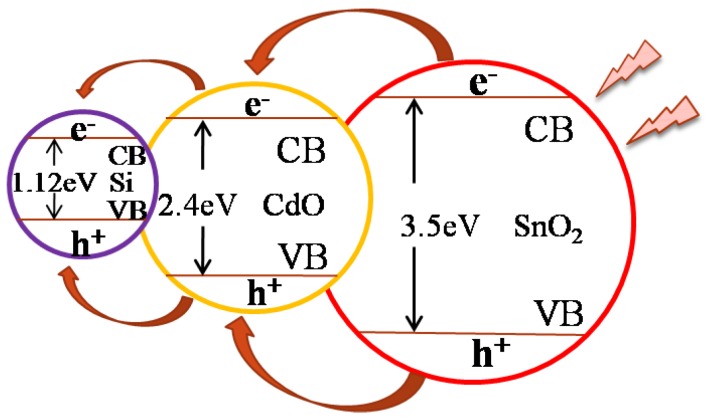
Schematic diagram representing the charge-transfer and electron–hole separation process in SnO_2_/CdO/Si DLSC.

To further investigate the mechanism of charge-transfer and electron–hole separation, we here illustrate the schematic diagram representing charge-transfer and electron–hole separation process in Scheme 1. As shown in the diagram, the conduction band (CB) of SnO_2_ lies at a more negative potential than that of CdO, while the valence band (VB) of CdO is more negative than that of SnO_2_. Under solar irradiation, photo-generated electrons in the conduction band of SnO_2_ go to the conduction band of CdO, and hole transfer occurs from the valence band of CdO to that of SnO_2_. At the same time, with a similar reason for c-Si substrate, electrons from conduction band of CdO transfer to that of c-Si, and holes transfer from valence band of CdO to that of c-Si. The simultaneous transfer of electrons and holes in SnO_2_/CdO/Si system increase both the yield and the lifetime of charge carriers by separating the photo-induced electrons and reducing charge recombination in the electron-transfer process [33]. In our research, the results of minority carrier lifetime, carrier density, and short current density are able to perfectly support the schematic diagram we discussed above.

**Figure 6:**
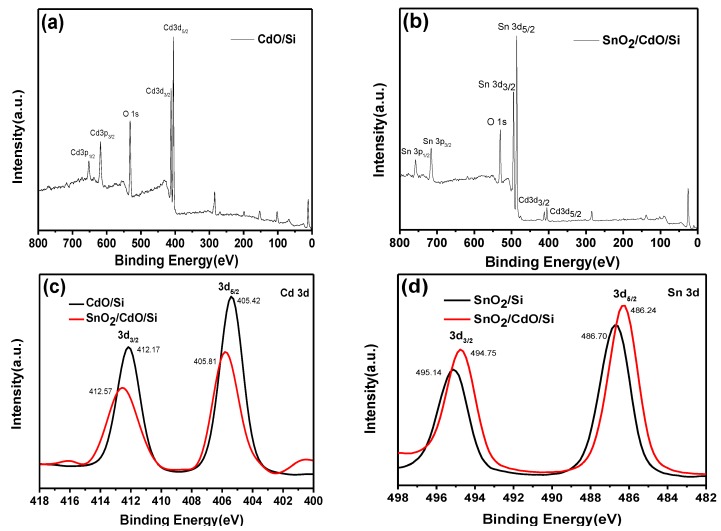
XPS survey spectra of (**a**) CdO/Si SLSC and (**b**) composited SnO_2_/CdO/Si DLSC. Core level (**c**) Cd 3d, (**d**) Sn 3d XPS spectra.

X-ray photoelectron spectroscopy (XPS) was also conducted to study the compositions and chemical states of as-synthesized SnO_2_/CdO/Si composited solar cell. Figure 6a,b compare the XPS survey spectrum of CdO/Si and SnO_2_/CdO/Si. In comparison to CdO/Si, the XPS survey spectrum of SnO_2_/CdO/Si exhibits four additional Sn peaks, and two strongest peaks refer to Sn 3d_3/2_ and Sn 3d_5/2_. When focusing on Cd 3d, we can find that peaks of Cd 3d in Figure 6b are apparently much weaker than that in Figure 6a. Because of a ~40 nm thickness SnO_2_ layer covered on top of CdO layer with spin-coating method, XPS signal of Cd was heavily blocked by SnO_2_ layer. The Cd 3d core level spectra of CdO/Si solar cell and SnO_2_/CdO/Si solar cell are shown in Figure 6c. For CdO/Si, peaks of Cd 3d center at 405.42 eV and 412.17 eV which are consistent with the values reported for Cd^2+^, while peaks of Cd 3d_5/2_ and Cd 3d_3/2_ are located at 405.81 eV and 412.57 eV respectively for SnO_2_/CdO/Si solar cell [34]. There is an approximate 0.4 eV increase in binding energy of Cd 3d between CdO/Si and SnO_2_/CdO/Si, which illustrates that the extraction of nuclei and electrons becomes stronger. It is believed that SnO_2_ crystalline and CdO crystalline are affected by each other and formed a kind of hetero-structure. This new formation of structure gives a vital impact on carrier-transport between different layers and also leads to a drastic improvement in the photovoltaic performance of SnO_2_/CdO/Si DLSC.

## 4. Conclusions

In conclusion, we investigated the photovoltaic performance of crystalline silicon solar cells using different metal oxide layers by band-gap alignment engineering that act as wavelength broadening layers for optical absorption and effective carrier separation and transport layers. The photovoltaic performance of as-synthesied SnO_2_/Si SLSC, CdO/Si SLSC, and SnO_2_/CdO/Si DLSC were considerably improved in comparison with original c-Si solar cells. The highest PCE value was 15.09% for SnO_2_/CdO/Si DLSC as measured while 12.28% for original c-Si solar cells. In addition, the recombination of photogenerated carriers was greatly restrained, resulting in a high minority carrier lifetime value. It is believed that by using band-gap alignment engineering, crystalline silicon solar cells still have deeper potential for further exploration.

## Figures and Tables

**Table 1:** Photovoltaic performance parameters of blank c-Si solar cell and as-synthesized CdO/Si and SnO_2_/Si SLSCs and SnO_2_/CdO/Si DLSC.

Solar Cell	*V_oc_* (V)	*J_sc_* (mA cm^−2^)	FF (%)	*η* (%)	Δ*η* (%)
c-Si	0.542	34.68	65.33	12.28	--
CdO/Si	0.556	36.12	66.42	13.34	8.6
SnO_2_/Si	0.565	37.01	66.76	13.96	13.6
SnO_2_/CdO/Si	0.575	38.20	68.70	15.09	22.8
